# Efficacy of Multivalent, Cochleate-Based Vaccine against *Salmonella* Infantis, *S.* Enteritidis and *S.* Typhimurium in Laying Hens

**DOI:** 10.3390/vaccines10020226

**Published:** 2022-02-01

**Authors:** Leonardo Sáenz, Miguel Guzmán, Sonia Vidal, Mario Caruffo, Daniela Siel, Caridad Zayas, Rodolfo Paredes, Carolina Valenzuela, Héctor Hidalgo, Oliver Pérez, Lisette Lapierre

**Affiliations:** 1 Faculty of Veterinary Sciences, Universidad de Chile, Santiago 8820808, Chile; leosaenz@uchile.cl (L.S.); mguzmanm@udla.cl (M.G.); svidal@vaccimed.cl (S.V.); mcaruffo@uchile.cl (M.C.); danisiel@uchile.cl (D.S.); caridadzayasv@gmail.com (C.Z.); cvalenzuelav@u.uchile.cl (C.V.); hhidalgo@uchile.cl (H.H.); 2 Núcleo de Investigaciones Aplicadas en Ciencias Veterinarias y Agronómicas, NIAVA, Facultad de Medicina Veterinaria y Agronomía, Campus Maipú–Sede Santiago, Universidad de las Américas, Santiago 9251454, Chile; 3 Escuela de Biotecnología, Facultad de Ciencias, Universidad Santo Tomás, Santiago 8370003, Chile; 4 Escuela de Medicina Veterinaria, Facultad de Ciencias de la Vida, Universidad Andres Bello, Santiago 8370035, Chile; rparedes@unab.cl; 5 Instituto de Ciencias Básicas Y Preclínicas “Victoria de Girón”, Universidad de Ciencias Médicas de La Habana, Havana 10600, Cuba; oliverperezmartin@gmail.com; 6 Lisette Lapierre, Faculty of Veterinary Sciences, University of Chile, Santiago 8820808, Chile

**Keywords:** *Salmonella* Infantis, vaccine, cochleate, ELISA, poultry

## Abstract

*Salmonella* enterica is an important foodborne pathogen. Commercial poultry are the main reservoirs of *Salmonella* enterica, leading to the contamination of food and outbreaks in humans. The vaccination of chickens is one of the most important strategies to reduce the number of *Salmonella* in poultry farms. Unfortunately, commercial vaccines have not been fully effective in controlling the spread and do not contain all the *Salmonella* serovars that circulate on farms. In this study, we evaluate a new, cochleate-based, trivalent injectable vaccine against *S.* Enteritidis, *S.* Typhimurium and *S.* Infantis, describing the vaccine security, capacity to induce specific anti-*Salmonella* serovar IgY and the gene expression of immune markers related to CD4 and CD8 T-cell-mediated immunity. Efficacy was evaluated through oral challenges performed separately for each *Salmonella* serotype. The efficacy and safety of the trivalent vaccine was proven under controlled conditions. The vaccine has no local or systemic reactions or adverse effects on poultry performance related to the vaccine. The vaccine provided significantly increased serum IgY titer levels, significantly reduced *Salmonella* CFU/g present in the cecum and an increased CD4+/CD8+ ratio in vaccinated animals when challenged with *S.* Infantis, *S.* Enteritidis and *S.* Typhimurium. These results indicate that this new trivalent vaccine does not generate adverse effects in poultry and produces an increase in neutralizing antibodies against the three *Salmonella* serovars.

## 1. Introduction

*Salmonella* enterica is an important gastroenteric pathogen, causing worldwide outbreaks in the human population. It is estimated that *Salmonella* enterica gastroenteritis is responsible for about 93.8 million illnesses and 155,000 deaths each year; of these, it is estimated that 80.3 million cases are foodborne, with very high associated costs [1]. Animal food products, especially eggs and poultry meat, have been the most common vehicles of *Salmonella* infections. Outbreaks might be caused by several serotypes of non-typhoidal *Salmonella* (NTS). *Salmonella* Enteritidis, *Salmonella* Typhimurium, *Salmonella* Newport, *Salmonella* Heidelberg and *Salmonella* Infantis are among the most important. *S.* enterica, serovar Enteritidis is implicated in 60% of salmonellosis in European people and is the world’s leading cause of salmonellosis. In the United States, *S.* Typhimurium is mostly associated with salmonellosis. In Europe, *S.* Infantis has emerged and is a frequently reported serovar from chicken meat (36.5%) and broilers (56.7%). In the USA, *S.* Infantis is commonly isolated from sick humans and poultry meat products. The European Union summary reported on trends and sources of zoonoses, zoonotic agents and food-borne outbreaks in 2016 [2,3]. In recent years, multi-drug-resistant *Salmonella* have emerged and complicate the treatment of at-risk groups. Drug resistance is prevalent in *S.* Typhimurium and *S.* Infantis serotypes and is successfully spread from broilers to humans through certain clones [4].

*Salmonella* is devastating to public health and has a negative economic impact on the poultry industry. To reduce the contamination of eggs and meat with *Salmonella* and thus prevent contamination in the human population, producing farms have comprehensive *Salmonella* control programs that include surveillance, biosecurity, management and vaccination [5]. Vaccination programs for poultry are frequently complex, involving multiple vaccinations with a range of different vaccines. Commercial *Salmonella* vaccines for poultry include live attenuated vaccines, inactivated (killed) vaccines and subunit vaccines and are typically based on strains of *S.* Enteritidis and *S.* Typhimurium. Multivalent vaccines are required to provide protection against the broad range of serovars found on poultry farms [6]. Subunit vaccines are safer because they only contain the antigens of the pathogen. However, multiple doses with these acellular fractions might be needed to confer long-lasting immunity against *Salmonella* [7]. One strategy to improve the immunogenicity of subunit vaccines is to use cochleate systems. Cochleates are phospholipid–calcium precipitates derived from the interaction of anionic lipid vesicles with divalent cations. They have a defined multilayered structure consisting of a solid, lipid bilayer sheet rolled up in a spiral [8]. The plasmatic membrane from *Salmonella* contains phospholipids that can be transformed into cochleates and several other molecules, such as proteins and lipopolysaccharides which can be used as a source of antigenic and immunogenic molecules [9,10].

The objective of our research was to study the safety and efficacy of a multivalent, subunit, cochleate-based vaccine against *S.* Enteritidis, *S.* Typhimurium and *S.* Infantis by vaccinating chickens in a controlled environment. Post-vaccination, chickens were separately challenged with virulent strains of each of the *Salmonella* serotypes. Cecum contamination and immune responses were analyzed.

## 2. Materials and Methods

### 2.1. Isolation and Serotyping of Salmonella Spp.

The *Salmonella* Enteritidis, *S.* Typhimurium and *S.* Infantis strains used were obtained through cloacal swabs from chickens belonging to commercial poultry flocks in the Metropolitan, Valparaíso and Libertador General Bernardo O’Higgins regions from Chile. The swabs were placed in sterile tubes containing 5 mL of Phosphate-Buffered Water (PBW) with Novobiocin (20 µg/mL) and incubated at 37 °C for 24 h. Tubes showing suspicious growth were sampled via streak-plate in XLD agar (Xylose lysine deoxycholate agar, Difco^®^, Merck, Kenilworth, NJ, USA), then incubated at 37 °C for 24 h. Samples that showed black or translucent colonies isolated via seeding on XLD agar were suspected to be *Salmonella* spp. The suspected *Salmonella* colonies were subjected to traditional morphological and biochemical testing including Gram staining and the use of triple sugar iron agar slopes and API 20E strips (bioMérieux, Marcy l’Etoile City, France). If two suspected colonies were confirmed as *Salmonella* spp., one of them was selected for a Polymerase Chain Reaction (PCR) to confirm the genus via the amplification of the *inv*A gene [11]. After biochemical and genotypic confirmation, *Salmonella* isolates were sent to the National Reference Laboratory (Institute Public Health, Santiago Chile) for serotype characterization using the Kauffman–White classification scheme [12].

### 2.2. Selection of the Strains of Each Salmonella Serotype to Be Used for the Vaccine Formulation

Six strains of each serotype were selected and analyzed for the following virulence genes: *gipA, trhH, spvC, sirA*, SEN1417, *pagK, sipA, mgtC* and prot6e via quantitative PCR (qPCR). The strains of each serotype that amplified the most of these virulence genes were evaluated for their ability to grow at different times in a broth at pH 5, as indicated by Choi and Groisman [13] and Retamal et al. [14]. One strain of each serotype with the highest OD at 24 h of growth at pH 5 (data not shown) was chosen for vaccine formulation.

### 2.3. Vaccine Preparation

A bacterial sediment was prepared from *S.* Enteritidis, *S.* Typhimurium and *S.* Infantis culture in broth Terrific (Merck^®^) with glycerol 0.5% *v*/*v* in a 15 L bioreactor (New Brunswick bioflo 415, Enfield, CT, USA) with automatically controlled conditions (oxygenation of 40%, pH of 7.0, temperature of 37 °C and agitation of 500 rpm) to obtain the highest yield of biomass. Bacteria were harvested via centrifugation, and the bacterial sediment was washed with sterile Phosphate-Buffered Saline (PBS) and was maintained in a frozen state until the membranes were purified. To purify the bacterial membrane fraction, bacterial sediment was suspended in a buffer containing 30 mmol/L of tris (hydroxymethyl) aminomethane (TRIS) at a ratio of 1:10 volume of bacterial sediment per TRIS. This suspension was continuously sonicated at 400 watts and 24 kHz (Hielscher Ultrasonic Processor UP400S; IU-P02, Germany). The suspension was added to 1.5% (*w*/*v*) sodium deoxycholate (DOC) and stirred at 150 rpm at 20 °C for 72 h and centrifuged at 250× *g* for 5 min and 15 °C. The supernatant was adjusted to a concentration of 5 mg of soluble protein/mL using the bicinchoninic acid method (BCA) (Merck, USA).

To induce calcium–cochleate formation, the bacterial membrane suspension was dripped into a solution containing 0.3% *w*/*v* Tris, 0.5% *w*/*v* NaCl and 0.2% *w*/*v* CaCl2 at pH 10. A wash solution was added to remove the DOC (containing 0.12% *w*/*v* Tris and 0.08% *w*/*v* NaCl at pH 10). Finally, the solution was centrifuged at 2900× *g* for 40 min at 4 °C, and the sediment with the cochleates was stored at 4 °C [10].

Every dose of vaccine was composed of 20 µg of each *Salmonella* serotype cochleate and 350 µL of Montanide™ (Paris, France) ISA 71 VG (ISA 71) as adjuvant and saline solution to a final volume of 500 µL as excipient. The vaccine formulations were made under GMP pilot plant conditions and were tested to assure quality regarding the following parameters: sterility, via microbiological culture; *Mycoplasma* spp. absence, using a MycoSensor qPCR Assay Kit (Agilent, Santa Clara, CA, USA); quantification of total protein using a BCA Protein Assay Kit (Merck); verification of specific protein production via sodium dodecyl sulfate polyacrylamide gel electrophoresis (SDS–PAGE); and pH determination using pH-indicator strips (Invitrogen, Waltham, MA, USA).

### 2.4. Characterization by Transmission Electron Microscopy (TEM) and SDS–PAGE

To obtain images via TEM, an aliquot of 10 µL of cochleate was deposited on grids (300 Mesh Formvar/Carbon 50/pk, Bussines Electronics SPA, Legnano, Italy) and stained with 1% *v*/*v* aqueous uranyl acetate for 1 min. Cochleates were observed using a transmission electron microscope (Philips Tecnai 12 BioTwin, FEI Company, Eindhoven, The Netherlands), operated at 80 kV. The photographs were processed through Megaview G2 Software. Five parts of the cochleates were mixed with one part of 6 × protein electrophoresis loading buffer (6 × Laemmli SDS Sample Buffer, Visual Protein), boiled for 5 min and run on a 15% SDS–PAGE denaturing gel. AccuRuler RGB PLUS Prestained Protein Ladder (AccuRuler RGB PLUS Prestained Protein Ladder) was used as a weight standard. Finally, the gel was stained with staining solution (0.1% *p*/*v* Coomassie Blue R-250, 7% *v*/*v* acetic acid and 40% *v*/*v* methanol).

**Figure 1:**
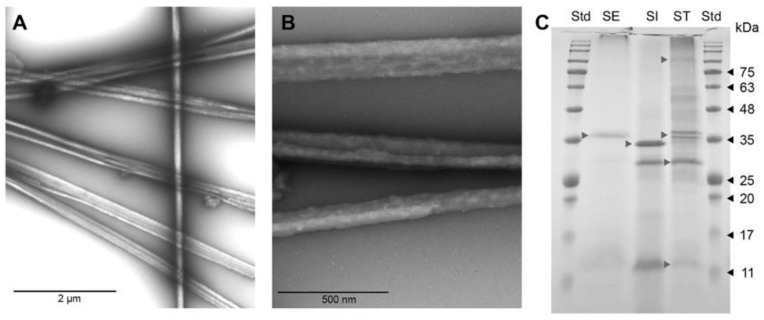
Transmission electron microscopy of cochleates derived from *Salmonella*-forming filamentous structures; (**A**) 9600× magnification, (**B**) 43,000× magnification, (**C**) SDS–PAGE 15% from cochleates formulated with *Salmonella* Enteritidis (SE), *Salmonella* Infantis (SI) and *Salmonella* Typhimurium (ST). Gray arrows show the main membrane proteins for each serotype. Black arrows indicate the molecular weights of AccuRuler RGB Plus Prestained Protein Ladder weight standard (Std) (Maestrogen, Taiwan, China). SDS–PAGE.

A 15% SDS–PAGE of the whole bacterial *Salmonella* serotypes was transferred to a nitrocellulose membrane and blocked with 5% *w*/*v* nonfat milk. They were incubated with serum from vaccinated animals or controls (day 35 of the assay), diluted 1:10,000 for 2 h at 37 °C, then incubated with HRP-bound chicken rabbit anti-IgY antibody (AbCam ab97140) 1:5000 for 45 min at 37 °C. Finally, the membranes were developed with 1-Step Ultra TMB-Blotting Solution (Thermo Scientific 37574, Waltham, MA, USA) (Figure 1 and Figure S1). In addition, lipopolysaccharide was measured through purpald analysis [15], while DNA content was measured using a spectrophotometer and agarose gel. These results are included as supplementary materials (Table S1).

### 2.5. Evaluation of Vaccine Safety and Adverse Effects

The safety of the trivalent parenteral vaccine was evaluated in 15 chickens on a single-dose schedule and 15 chickens on a triple-dose schedule. These white leghorn females were received at one day of age from a commercial hatchery, reared until six weeks of age, when they were separated into two groups (single-dose and triple-dose) and inoculated once in the breast muscle. The animals were followed for 15 days. Behavioral, physiological and histological parameters were measured to determine vaccine safety. Chickens were observed daily, and any change was scored as presented in Table 1, which is modified from the Guidelines on the recognition of pain, distress, and discomfort in experimental animals and a hypothesis for assessment [16].

If scores in a trial group reached 6–9, the observation frequency would be doubled and environmental parameters verified. However, if the score reached 10–12, the experiment would be terminated, the birds would be euthanized, and experimental conditions would be re-evaluated before performing a new assay. At the end of the experiment, all animals were euthanized, and a necropsy was performed for histopathology in different tissues.

### 2.6. Evaluation of Vaccine Efficacy

Three independent vaccination/challenge experiments were carried out, as summarized in Table 2. One-day-old *Salmonella*-free white Leghorn chickens were received at the Avian Pathology Laboratory (Universidad de Chile) from a commercial hatchery. *Salmonella* spp. absence was verified via feces culture. Chickens were reared according to genetic line parameters until six weeks. Three different vaccination and challenge experiments were performed with the three *Salmonella* serotypes. Chickens were randomly separated into 2 groups of 14 chickens according to Wilde et al. [17] and the available space in the community cages inside different isolated rooms. The groups were: vaccinated group (VG) or only adjuvant control group (CG). At 6 weeks, the vaccine or adjuvant was administered into the breast muscle via intramuscular injection. At nine weeks, chickens received a second dose. At ten weeks, the chickens were deprived of food and water overnight (12 h) and the next morning, i.e., one week after the booster vaccination [18], they were orally infected with 10^9^ colony-forming units (CFUs) of a *Salmonella* serotype suspended in 0.5 mL of PBS. All animals were challenged, both control and vaccinated groups. For each challenge, an oral gavage needle was used to avoid inoculum losses. All chickens were challenged with each *Salmonella* serovar, which were administered one at a time. The experiment finished at 11 weeks, the final samples of cecum and blood were taken, and the chickens were euthanized via cervical dislocation. Blood samples were taken from the brachial vein of each bird prior to vaccination and one week after the challenge. In addition, a sample of litter was taken prior to vaccination and challenge from each floor pen to screen for the presence of environmental *Salmonella*. This evaluation of the presence of environmental Salmonella was performed via conventional microbiological culture, based on the standard ISO 6579:2017. Throughout the experiment, the chickens had food according to nutrient requirements and water ad libitum. Only authorized personnel were allowed to manipulate the chickens.

### 2.7. Antibody ELISA

**Figure 2:**
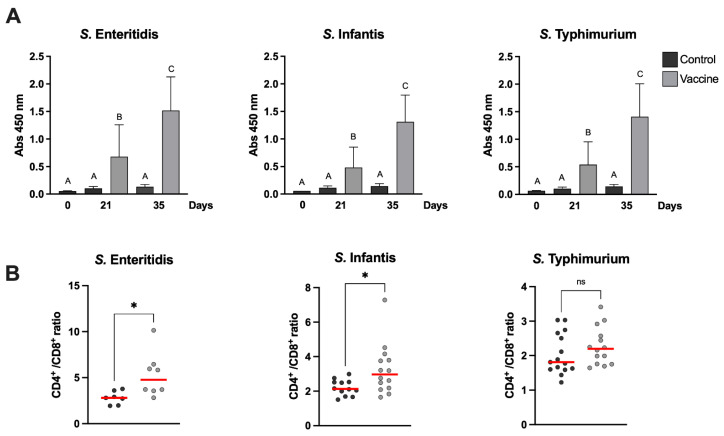
Effect of vaccines on the level of serum-specific *Salmonell*a IgY and flow cytometry analyses of CD8+/CD4+ cell ratio. (**A**) IgY quantification in immunized or control chickens on days 1 and 21 after first vaccination. Data show mean ± SD of 14 chickens per group. The Kruskal–Wallis test followed by Dunn’s post-test were performed; different letters represent significant differences (*p* ≤ 0.05). (**B**) Peripheral T lymphocytes were immunostained, and the frequency of CD3+CD4+ and CD3+CD8+ lymphocytes in blood was determined. Figure shows the CD4+/CD8+ ratio in the vaccinated and control groups, 7 days after challenge. Red lines correspond to the median of each group and asterisks show significant differences (*p* ≥ 0.05) obtained by unpaired *t*-test.

Determination of serum IgY levels against specific antigen from *Salmonella* serotypes were measured using an indirect ELISA on day 0 and days 21 and 35 after first vaccination (Table 2). Serum was separated from blood samples via centrifugation. Then, 96-well Polysorp plates (Nunc, Thermo Scientific, Waltham, MA, USA) were coated with 2 µg of whole *Salmonella*, which were frozen and thawed before use, in each well with 50 µL of coating buffer (150 mM Na_2_CO_3_, 350 mM NaHCO_3_, pH 9.6) overnight at 4 °C. Then, the plates were washed with washing buffer (0.02% Triton × 100 *v*:*v* in PBS) and blocked with 200 µL of blocking buffer (5% *w*:*v* skim milk in PBS) for 2 h at 37 °C. Subsequently, the plates were incubated with 100 µL of diluted 1:10000 serum in diluent buffer (0.5% skim milk *w*:*v*, 0.02% Triton × 100 *v*:*v* in PBS) for 2 h at 37 °C. Plates were then washed and incubated with 100 µL of diluted rabbit anti-Chicken IgY peroxidase-conjugated antibodies (1:5000) for 1 h at 37 °C. The plates were washed and 100 µL of TMB substrate solution was added (1-Step Ultra TMB-ELISA, Pierce) for 2.5 min at room temperature, and the reaction was stopped with 100 µL of 2N sulfuric acid. Finally, the absorbance was measured at 450 nm (Figure 2A).

### 2.8. Flow Cytometric Phenotyping of Peripheral Lymphocytes

To examine the effective induction of a T lymphocyte response upon vaccination, peripheral blood samples were collected from each animal after they were euthanized (7 days after bacterial challenge, Table 2). Lymphocytes from peripheral blood were purified by Ficoll-Paque plus (GE Healthcare). Briefly, 2 mL of the whole blood in 2 mL of PBS with 3 mL of Ficoll-Paque were centrifuged at 400 *g* for 40 min at 18 °C. The lymphocyte layer (whitish phase) was washed in PBS and resuspended in 200 µL of 1 × PBS.

The frequency of helper (CD3+CD4+) and cytotoxic (CD3+CD8+) lymphocytes in vaccinated and control groups were evaluated via flow cytometry. For surface labeling of the different lymphocyte populations, 1 µL of anti-CD3+ (PE), anti-CD4+ (FITC) and anti-CD8+ (PeC5) antibodies were added and incubated for 70 min at 4 °C in the dark. Samples were centrifuged at 500 *g* for 5 min. The supernatant was discarded and resuspended in 2 mL of Focusing buffer (Invitrogen). Analysis was performed using a Attune NxT Flow Cytometer and for compensation, the AbC™ Total Antibody Compensation Bead Kit (Invitrogen) was used according to the manufacturer’s instructions. The results were expressed as the CD4+/CD8+ cell ratio, which represents the ratio between CD3+CD4+ and CD3+CD8+ lymphocytes. The results are shown Figure 2B

### 2.9. Bacterial Counts in the Cecum of Poultry

**Figure 3:**
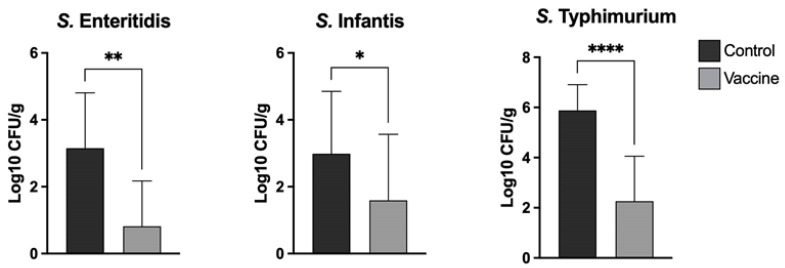
Efficacy of vaccination in reducing bacterial. Chickens were orally challenged at 10 weeks of age with 10^9^ CFU of each *Salmonella* serotype and seven days later, the challenged serotype was quantified via plate culture in the cecum. Data show mean ± SD of 14 chickens per group. An unpaired *t*-test was performed; asterisks show statistical significance: * *p* ≤ 0.05, ** *p* ≤ 0.01, **** *p* ≤ 0.0001.

For the determination of cecum colonization of the *S.* Enteritidis, *S.* Typhimurium and *S.* Infantis strains, 7 days after challenge (35 days after first vaccination, Table 2), all chickens were euthanized. Cecum was collected under aseptic conditions, weighed and homogenized on stomacher bags prior to dilution in 5 mL of Buffered Peptone Water (BPW). Suspensions were 10-fold diluted up to 10 × 10^−5^ and aliquots were plated onto XLD agar as appropriate for bacterial counts. The plates were incubated at 41°C for 18 to 24 h, and the number of *Salmonella* obtained via direct culture was counted and recorded. Log10 units of protection were obtained by subtracting the mean Log10 CFU for each experimental group from the mean Log10 CFU of the control group. For samples where no colonies were detected, a value of 1 CFU was used, thus yielding a sample-specific detection limit. The results are shown Figure 3.

### 2.10. Statistical Analysis

Statistical analysis was performed using the software GraphPad Prism 8 (Graphpad Software, Inc). Differences in antibodies were analyzed using the Kruskal–Wallis test followed by Dunn’s post-test for multiple comparisons. Differences in bacterial burden and immunophenotyping of peripheral blood lymphocytes were analyzed using an unpaired *t*-test. *p* ≤ 0.05 was considered significant, and all experiments were performed at least in triplicate.

## 3. Results

### 3.1. Transmission Electron Microscopy (TEM) and SDS–PAGE

When observed using TEM, the cochleates showed a highly conserved tubular morphology of several micrometers long but only a few nanometers in diameter, as shown in Figure 1A,B. As shown in Figure 1C, the electrophoretic pattern of the antigens retained in the cochleates is different for each *salmonella* serotype, being notorious in the serotype Infantis. The protein concentration in the cochleates was 1 to 2 mg/mL.

### 3.2. Evaluation of Safety and Adverse Effects of the Vaccine in Chickens

According to the scoring system described in Table 1, no altered parameters were observed in the control or treatment group. The birds were euthanized fifteen days post-vaccination in the safety trial and one-week post-challenge in the efficacy assays. Necropsies were performed on every bird. No alterations were detected at the injection site; in the safety trial vaccine, remnants could be observed without lesions associated. No macroscopic lesions were found in the tissues and organs evaluated, and all were classified as score 0, which indicates normal. Therefore, no differences were observed between groups. No change was exhibited in tongue, esophagus, crop, proventriculus, gizzard, small and large intestines, as well as the main organs. The intestinal lumen was evaluated for changes in wall integrity or mucosal face, but no lesions were found. During the trial, the birds were alert to the environment and to manipulation and were active at a level expected for their age. No deterioration in general health or reduction in feed intake was seen during the trials throughout the whole observation period. The vaccine formulations proved to be safe and did not produce adverse reactions in the chickens.

### 3.3. Specific ELISA against Salmonella Serotypes

To determine the capacity of the trivalent vaccine to induce specific antibodies against the three *Salmonella* serotypes (*S*. Enteritidis, *S.* Infantis and *S.* Typhimurium), IgY induction in plasma was quantified via ELISA. No specific antibodies were detected in the control groups at day 21 or 35 post-vaccination (day 21 post-vaccination corresponds to 9 weeks age of the bird and day 35 post-vaccination to 11 weeks age), unlike the vaccinated groups which showed a significant difference in relation to the control at both days (21 and 35) for the three serotypes evaluated. When comparing antibody levels between time points within the same serotype, significant changes were observed between days with an increase in the induction of antibodies at day 35 (Figure 2A). We can conclude that the trivalent *Salmonella* vaccine is able to induce specific antibodies by 21 days post-vaccination, supported by Western blot results, included as supplementary materials.

### 3.4. Immunophenotyping of Peripheral Blood Lymphocytes

As shown in Figure 2B, the CD4+/CD8+ ratio was significantly higher in vaccinated than in control animals when challenged with *S.* Infantis and *S.* Enteritidis (*p* ≥ 0.05). In contrast, when challenged with *S.* Typhimurium, no statistically significant differences were observed between the vaccinated and control group (*p* ≤ 0.05).

### 3.5. Environmental Monitoring Salmonella Spp.

No extraneous *Salmonella* were detected before challenge in all experiments, and in all groups, by environmental monitoring of litter, providing further confirmation that the birds had not been subject to prior *Salmonella* infection.

### 3.6. Experimental Challenge

Challenge trials with each of the *Salmonella* serotypes were performed on 10-week-old chickens. Following challenge, no clinical symptoms were observed with any of serovars tested. In the vaccinated group, there was a statistically significant reduction in the number of *Salmonella* recovered in ceca samples compared with the control group in each challenge with the three serovars (Figure 3). In both the cecum of unvaccinated and vaccinated chickens, the prevalence of colonization after challenge with *Salmonella* serovar Typhimurium was higher compared to the other two serovars. Vaccinated groups showed a significant decrease in bacterial counts compared to the control groups, with reductions of 2.33 (73.9%), 1.39 (46.6%) and 3.61 (61.4%) Log10 CFU/g for *S.* Enteritidis, *S.* Infantis and *S.* Typhimurium, respectively (Figure 3). The increase in the concentration of specific antibodies at day 35 match with an effective decrease in the bacterial loads of these serotypes in the intestine of vaccinated individuals. This suggests a correlation between these variables that needs to be addressed in future studies. We also determined the *S.* Enteritidis, *S.* Infantis and *S.* Typhimurium count in spleen and liver for those groups. However, the bacterial counts were lower overall with no significant difference between groups (data not shown).

## 4. Discussions

*Salmonella* contamination in poultry food products is a very important problem for both public health and the industry. The most important *Salmonella* serotypes associated with poultry and that cause disease in humans are *S.* Enteritidis and *S.* Typhimurium and, consequently, most vaccines available for administration in poultry contain one or both serotypes. However, other zoonotic *Salmonella* serotypes have emerged in recent years and there are no commercial vaccines against them. The emergence of the *S.* Infantis serotype has been described in European countries and the United States [4,19,20]. In South America, S. Infantis has also been described in Ecuador [21], Perú [22] and Chile [23]. In Brazil, *S.* Infantis has been catalogued as the second most prevalent serotype in broilers [24]. The vaccination of breeder-pullets, along with other intervention measures, is an important strategy that is currently being used to reduce the levels of *Salmonella* in poultry flocks, which will ultimately lead to lower rates of human *Salmonella* infections [6]. For this reason, it is essential that the available vaccines cover all the *Salmonella* serotypes present or most frequently found in poultry farms. This study investigated the efficacy and safety of a new trivalent, subunit, cochleate-based vaccine against *S.* Enteritidis, *S.* Typhimurium and *S.* Infantis to be administered parenterally in laying hens.

Our subunit vaccine design utilizes an enriched extract of solubilized bacterial membranes, which upon exposure to a CaCl2 solution coiled into filamentous cochlear structures of nanometer diameters, stabilized by calcium atoms. The coiled membranes capture and retain a wide range of bacterial antigens, both membrane and cytoplasmic proteins, together with traces of immunostimulatory molecules such as lipopolysaccharide or LPS, bacterial DNA and RNA, etc. These pathogen-associated molecular patterns (PAMP) are conserved structures that bind to specific receptors on immune system cells. Lipopolysaccharide, for example, present in Gram-negative bacteria, stimulates the immune response through the binding and activation of Toll-like receptor 4, together with lipopolysaccharide binding protein (LBP), CD14 and MD-2, on antigen-presenting cells. LPS induces the expression of proinflammatory cytokines such as Il1b and TnFa, co-stimulatory molecules (CD40, CD80 and CD86) and cytokine secretion (IL12, IL 2), which increases antigenic presentation to CD4+ LTs, and consequently, the adaptive immune response. LPS has a detrimental effect as an endotoxin is due to the hyperactivation of TLR4 that occurs when present in high amounts; however, in our formulation, it is trapped in cochleates, which favor a long-term protective response [25].

Outer-membrane protein vaccines with adjuvants have been used to decrease the shedding of *S.* Enteritidis in poultry. It has been reported that immunized 9-week-old chickens with two outer-membrane proteins subcutaneously, followed by two boost immunizations with time intervals of 2 weeks, decreasing cecal colonization about 1000-fold when the animals were infected orally with 8 × 10^8^ CFU of a virulent *S.* Enteritidis strain [26,27]. Additionally, a subunit vaccine has been evaluated against the de-flagellated or SEp9 antigen of *S.* Enteritidis, which decreases the bacterial colonization of the chicken intestine [27,28]. In another study, Desin et al. [29] evaluated a subunit vaccine based on the type 1 pathogenicity island (SPI-1 T3SS), which resulted in high antibody titers and lead to a reduction in the levels of *S.* Enteritidis in the liver, but not in the spleen and ceca. Li et al. [18] experimentally evaluated a subunit vaccine against *S.* Enteritidis in poultry to provide protection against *Salmonella* Enteritidis challenge in chickens, which induced strong immune responses.

Interestingly, our study shows that the electrophoretic profile of the proteins retained in the cochleae for the three *Salmonella* serotypes is different, and therefore, we expanded the range of surface antigens present in the formulation (Figure 1C). The outer-membrane proteins (OMPs) of *Salmonella* represent important virulence factors as well as being highly immunogenic [30]. In contrast to the use of a single antigen, the use of a varied set of antigens for each serotype broadens the spectrum of specific antibodies generated by immunized animals.

The antigenic component of non-live vaccines can be killed whole organisms, purified proteins from the organism, recombinant proteins or polysaccharides [31]. Our polyvalent vaccine based on cochleates is well characterized and contains outer-membrane proteins (OMP), LPS and bacterial DNA. As it was observed in Figure 1C, the pattern of proteins present in each cochleate is constant and can be compared with the electrophoretic pattern of the master seeds for each Salmonella serotype from Figures S1 and S2. The presence of LPS and bacterial DNA was also quantified, as shown in Table S1. No specific antibodies against any Salmonella serotype were observed in the animals before the vaccination, demonstrating that maternal antibodies are no longer present at 6 weeks of age. Three weeks after the first vaccination, there was already a significant increase in specific antibodies against *Salmonella* in all vaccinated animals; this increase in antibodies persisted until day 35 post first vaccination, which was the last day of sampling in our experiment (Figure 2A). The presence of immunostimulatory molecules in the cochleae, such as LPS, favors the immune response not only with an increase in antibody levels but also in the progression of the T-lymphocyte-dependent immune response; in fact, we could see a functional B- and T-cell response in the vaccinated animals before and after the challenge.

CD4+ T lymphocytes play a major role in protective immunity during primary and secondary *Salmonella* infection. CD4+ helper T cells release cytokines such as IL-2 and IFNγ to further stimulate NK cells, macrophages and CD8+ cytotoxic T cells and promote the differentiation of B cells into antibody-producing plasma cells [32,33,34]. IFNγ has been reported to activate macrophages, resulting in the improved clearance of engulfed bacteria. It has been shown that decreased activity of peritoneal macrophage is associated with the increased systemic dissemination of *S.* Enteritidis in chickens. In addition, *Salmonella* is known to be resistant to clearance by macrophages and to even use macrophages as support for systemic dissemination [32]. We were able to determine that after a challenge with two of the three *Salmonella* serotypes (*S.* Enteritidis and *S.* Infantis), there was a significant increase in the CD4+/CD8+ ratio in vaccinated chickens (Figure 2B). In the case of the group challenged with *S*. Typhimurium, although the median CD4+/CD8+ ratio was higher in the vaccinated group compared to the control, no significant differences were observed, possibly due to the large dispersion of the data obtained in this group (Figure 2B). The post hoc statistical power of the analyses was calculated for each serovar against its control; they were 0.972 (*S.* Typhimurium), 0.999 (*S.* Enteritidis), and 1 (*S.* Infantis). Thus, the non-statistical difference observed with *S.* Typhimurium is unrelated to the size of the group.

Recently, [35] described the changes in the innate and adaptive immune system that are generated in birds after infection with *S.* Enteritidis in detail. As in the control group of our study, one week after the challenge with *Salmonella*, there was an increase in the proportion of CD8+ T lymphocytes and other cells, but this, according to these authors, is not sufficient to prevent the progressive colonization of the cecum and spleen with *Salmonella.* However, in our study, the vaccine was able to significantly decrease *Salmonella* colonization of the cecum in all challenged vaccinated chickens.

The chickens were challenged separately with a high infecting dose of each *Salmonella* serotype present in the vaccine. The bacterial burden was quantified in the cecum seven days post-challenge. The results of our study show that the trivalent vaccine was able to significantly reduce bacterial colonization with each of the serovars tested (Figure 3). The reduction in the number of UFC in the assays was highest for serovar *S.* Typhimurium followed by serovar *S.* Enteritidis and serovar *S.* Infantis. Interestingly, the levels of spleen and liver invasion of the three *Salmonella* serotypes were very low in all the animals sampled, both in the control and vaccinated groups, with the latter showing no detectable bacterial growth in any sample. This may be due to the fact that these *Salmonella* serovars are zoonotic and do not produce disease in chickens. This is in agreement with the observations of Berndt et al. [36], who found *S.* Infantis to only have limited capability to invade the lamina propria in very young chickens. Considering the UFC reduction in all vaccinated groups against their respective controls, new power analyses were performed; in all analyses, the power calculated was equal to 1.

In contrast to the currently used live attenuated, commercial vaccines, the developed vaccine possesses antigens of all three Salmonella serotypes, including the currently relevant Infantis serotype. Moreover, unlike inactivated vaccines in which the antigens are denatured by formalin treatment, the antigens captured in the cochleates are preserved together with immunostimulatory molecules that induce a CD4+ LT-mediated response. This, together with a reduced production cost, enhances the novelty of this new type of formulation. In summary, our parenterally administered subunit trivalent vaccine based on cochleates was effective and did not show any adverse effect in the birds in our controlled environment trials. The doses of bacteria delivered in the challenge trials (1 × 10^9^ CFU/mL) were generally higher than the *Salmonella* infection pressure to which birds are naturally exposed to in commercial farms, and our vaccine was able to significantly decrease the number of *Salmonella* colonizing the caecum in vaccinated birds compared to control birds.

## 5. Conclusions

The trivalent vaccine against *Salmonella* Enteritidis, *S.* Typhimurium and *S.* Infantis was safe and effective when administered parenterally in two doses in chickens. In the safety studies, the vaccine did not cause any adverse effects in the animals, and when the efficacy study was performed, the vaccine was able to increase specific antibodies that react against the three *Salmonella* serovars and induce a cellular immune response mediated by CD3+CD4 lymphocytes. Immunization significantly decreased the intestinal colonization of virulent strains in vaccinated chickens. These preliminary data suggest that this trivalent, cochleate-based vaccine could be administered in commercial layer flocks during the rearing stage. This study opens a new line of research regarding this vaccine; new field trials should be performed in order to demonstrate the safety of commercial eggs, as well as the protection of chickens through maternal antibodies.

## Figures and Tables

**Table 1:** Signs score.

Parameters	Signs	Score
Weight loss	No alterations	0
	Weight loss below 10%	1
	Weight loss between 10–20%	2
	Weight loss greater than 20%	3
Aspect	No alterations	0
	Ruffled feathers	1
	Ruffled feathers + wings and tail dropped	2
	Ruffled feathers + wings and tail dropped + dirty tail	3
Behavior	No alterations	0
	Feeding activity decreased (observed at feeding time)	1
	Careless of environment, sagging through their legs to sitting position	2
	Depressed birds/birds with stupor	3
Vital signs	No alterations	0
	Increment up to 2 °C in temperature	1
	Increment greater than 2 °C in temperature	2
	Previous signs + change in cardiac and respiratory frequency	3

**Table 2:** Summary of experimental design for each vaccination challenge experiment.

Experiment No	Group Size CG/VG	Vaccination Schedule Weeks(Day)	Challenge Serovar	Challenge Dose CFU/Bird	Age at Challenge Weeks	Blood Sampling Weeks (Post-Vaccination Day)	Cecum Sampling Weeks (Post-Vaccination Day)
1	14/14	6 (1) and 9 (21)	*S*. I *	1 × 10^9^	10	6 (1),9 (21) and 11 (35)	11 (35)
2	14/14	6 (1) and 9 (21)	*S*. E *	1 × 10^9^	10	6 (1),9 (21) and 11 (35)	11 (35)
3	14/14	6 (1) and 9 (21)	*S*. T *	1 × 10^9^	10	6 (1),9 (21) and 11 (35)	11 (35)

*S*. I *: *Salmonella* Infantis; *S*. E *: *Salmonella* Enteritidis; *S*. T *: *Salmonella* Typhimurium.

## Data Availability

The data presented in this study are available on request from the corresponding author.

## Supplementary Materials

The following are available online at https://www.mdpi.com/article/10.3390/vaccines10020226/s1, Table S1: Characterization of immunostimulatory elements in vaccine formulations. Figure S1. SDS-PAGE and Western blot analysis of cross-reactivity between serum from vaccinated and control groups against *S.* Enteritidis (SE), *S.* Infantis (SI), *S.* Typhimurium (ST) at 35 days post-vaccination. A. Results of SDS-PAGE showing the protein profile of SE, SI, ST. B. Results of IgY Western blot analysis using 1:10,000 diluted serum from vaccinated chickens with the trivalent vaccine at 35 days post-vaccination. C. Results of IgY Western blot analysis using 1:10,000 diluted serum from control non-vaccinated chickens. Molecular size standards (in kilodaltons) are given on the left. Figure S2. SDS-PAGE and Western blot densitometric analysis for the three Salmonella serotypes band profile. Line 1: AccuRuler RGB PLUS Prestained Protein Ladder (Maestrogen), Line 2: *S.* Enteritidis, Line 3: *S.* Infantis, Line 4: Typhimurium. (A) SDS-PAGE under reducing conditions and densitometric scans with the band profile of the different Salmonella serotypes. Blotted membranes reacted with using sera from immunized animals with multivalent vaccines (B) and unvaccinated control (C). Densitometric analysis was performed using software Gelpro 3.1 and tables show the relative percentage of each band in relation to the total of that line.
