# Supplementary material for: Efficacy of Multivalent, Cochleate-Based Vaccine against Salmonella Infantis, S. Enteritidis and S. Typhimurium in Laying Hens
Source: Vaccines (Basel). 2022 Feb 1;10(2):226. doi: 10.3390/vaccines10020226 (PMC8879397; doi:10.3390/vaccines10020226)
Supplement: Supplementary file 1 [file vaccines-10-00226-s001.zip › vaccines-1434309-supplementary.pdf]

Supplementary

	S. **Enteritidis**	*S.* **Infantis**	*S.* **Typhimurium**
DNA content (ng/µl)	740.11 ± 21.77	1848.16 ± 13.61	1192.19 ± 50.74
LPS content* (UE / mg)	177805.1 ± 11430.3	24648.62 ± 892.33	80831.33 ± 4982.34

Table S1. Characterization of immunostimulatory elements in vaccine formulations.

* Endotoxin units per mg of protein present in cochleates.

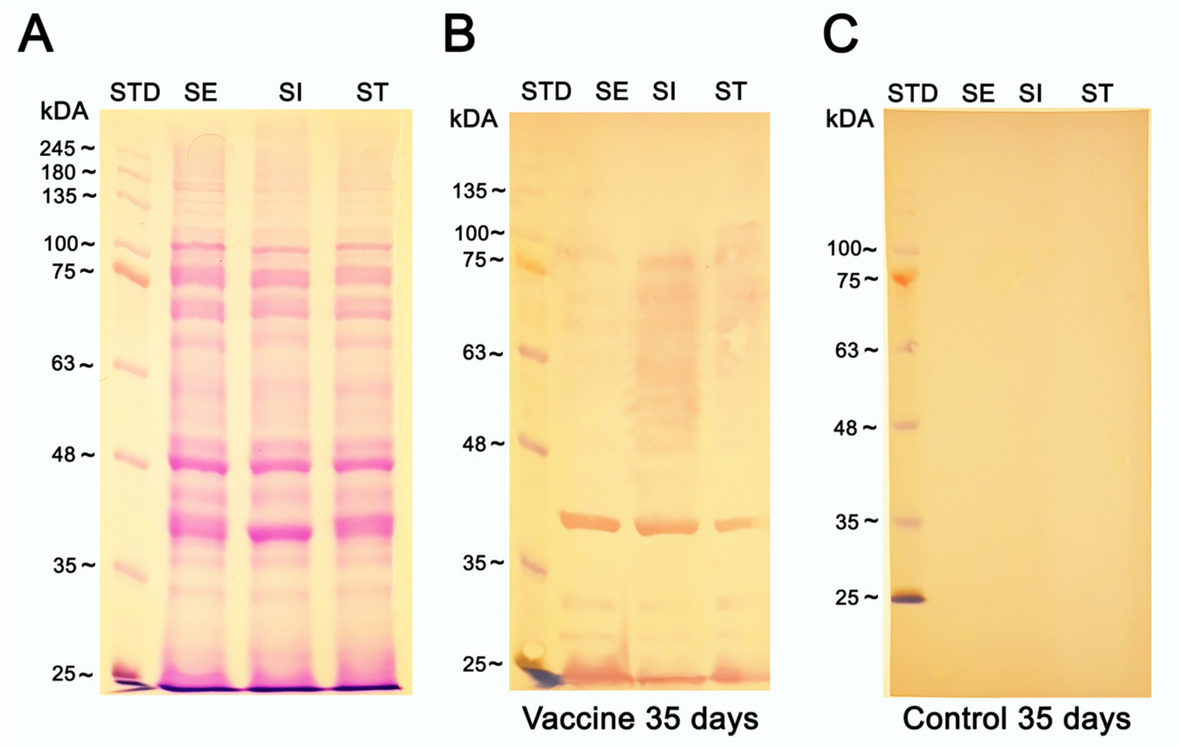

Figure S1. SDS-PAGE and Western blot analysis of cross-reactivity between serum from vaccinated and control groups against *S.* Enteritidis (SE), *S.* Infantis (SI), *S.* Typhimurium (ST) at 35 days post-vaccination. A. Results of SDS-PAGE showing the protein profile of SE, SI, ST. B. Results of IgY Western blot analysis using 1:10.000 diluted serum from vaccinated chickens with the trivalent vaccine at 35 days post-vaccination. C. Results of IgY Western blot analysis using 1:10.000 diluted serum from control non-vaccinated chickens. Molecular size standards (in kilodaltons) are given on the left.

A

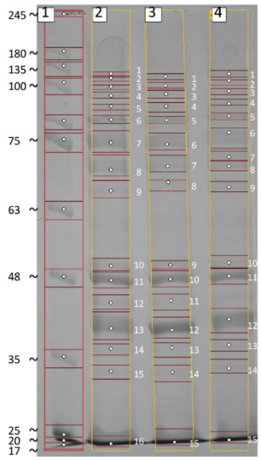

GEL SECTION	DENSITOMETRY %		
	**2**	**3**	**4**
**1**	0.4	0.7	0.7
**2**	0.4	0.4	0.3
**3**	0.4	0.6	0.6
**4**	0.5	0.6	0.6
**5**	0.6	2.9	3.7
**6**	3.7	8.0	8.3
**7**	10.8	4.1	1.2
**8**	7.6	1.4	3.3
**9**	2.9	1.2	1.4
**10**	1.7	13.7	1.5
**11**	14.6	1.8	16.8
**12**	3.0	24.8	23.5
**13**	21.6	1.0	1.2
**14**	1.3	1.7	1.6
**15**	1.8	37.1	35.3
**16**	28.7		

C

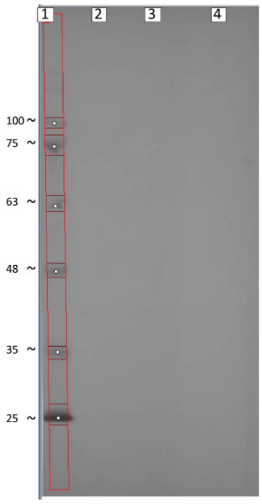

B

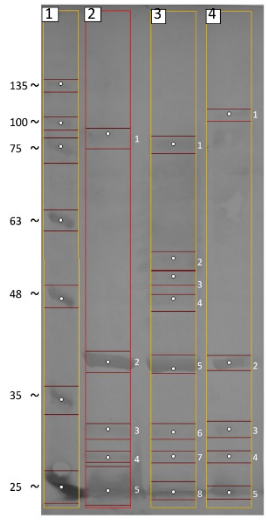

GEL SECTION	DENSITOMETRY %		
	**2**	**3**	**4**
**1**	6.2	11.0	6.0
**2**	30.8	6.4	43.1
**3**	4.3	4.0	13.4
**4**	3.5	4.5	5.0
**5**	55.3	41.2	32.5
**6**		3.2	
**7**		2.8	
**8**		26.8	

Figure S2. SDS-PAGE and Western blot densitometric analysis for the three Salmonella serotypes band profile. Line 1: AccuRuler RGB PLUS Prestained Protein Ladder (Maestrogen), Line 2: S. Enteritidis, Line 3: S. Infantis, Line 4: Typhimurium. A) SDS-PAGE under reducing conditions and densitometric scans with the band profile of the different Salmonella serotypes. Blotted membranes reacted with using sera from immunized animals with multivalent vaccines (B) and unvaccinated control (C). Densitometric analysis was performed using software Gelpro 3.1 and tables show the relative percentage of each band in relation to the total of that line.
